# The disordered C terminus of ALKBH5 promotes phase separation and paraspeckles assembly

**DOI:** 10.1016/j.jbc.2023.105071

**Published:** 2023-07-18

**Authors:** Xiaoyang Qin, Yan Long, Xue Bai, Lei Cao, Han Yan, Kai Zhang, Bo Wang, Xudong Wu

**Affiliations:** 1 State Key Laboratory of Experimental Hematology, The Province and Ministry Co-Sponsored Collaborative Innovation Center for Medical Epigenetics, Key Laboratory of Immune Microenvironment and Disease (Ministry of Education), Tianjin Key Laboratory of Medical Epigenetics, Department of Cell Biology, Tianjin Medical University, Tianjin, China; 2 Department of Biochemistry and Molecular Biology, Tianjin Medical University, Tianjin, China; 3 Department of Neurosurgery, Tianjin Huanhu Hospital, Tianjin Key Laboratory of Cerebral Vascular and Neurodegenerative Diseases, Tianjin Neurosurgical Institute, Tianjin, China; 4 Department of Neurosurgery, Tianjin Medical University General Hospital, Tianjin, China

**Keywords:** ALKBH5, paraspeckle, phase separation, NEAT1, m^6^A

## Abstract

Paraspeckles (PS) are nuclear structures scaffolded by the long noncoding RNA NEAT1 and protein components such as NONO and SFPQ. We previously found that the upregulation of RNA N6-methyl-adenosine (m^6^A) demethylase ALKBH5 facilitates hypoxia-induced paraspeckle assembly through erasing m^6^A marks on NEAT1, thus stabilizing it. However, it remains unclear how these processes are spatiotemporally coordinated. Here we discover that ALKBH5 specifically binds to proteins in PS and forms phase-separated droplets that are incorporated into PS through its C-terminal intrinsically disordered region (cIDR). Upon exposure to hypoxia, rapid ALKBH5 condensation in PS induces m^6^A demethylation of NEAT1, which further facilitates PS formation before the upregulation of ALKBH5 expression. In cells expressing ALKBH5 lacking cIDR, PS fail to be formed in response to hypoxia, accompanied with insufficient m^6^A demethylation of NEAT1 and its destabilization. We also demonstrate that ALKBH5-cIDR is indispensable for hypoxia-induced effects such as cancer cell invasion. Therefore, our study has identified the role of ALKBH5 in phase separation as the molecular basis of the positive feedback loop for PS formation between ALKBH5 incorporation into PS and NEAT1 stabilization.

Paraspeckles (PS) are one of subnuclear structures found in the interchromatin space. They appear as discrete puncta and are composed of long noncoding RNA NEAT1 (nuclear paraspeckle assembly transcript 1) and protein components such as P54NRB/NONO, PSPC1, and PSF/SFPQ (1, 2, 3, 4). NEAT1 is an essential component of PS, and its deletion leads to the disintegration of these structures. Actually, NEAT1 has two isoforms in mammals: NEAT1-1 (3.7 kb) and NEAT1-2 (22.7 kb). The PS proteins NONO and SFPQ specifically associate with the middle domain of NEAT1-2, which stabilizes this transcript, making it critical for the structure and maintenance of PS (5, 6).

Paraspeckles are dynamically assembled and disassembled during development and in response to various stresses (4, 7, 8). However, the precise regulatory mechanisms governing these dynamic processes remain incompletely understood. While posttranslational modification of PS proteins (9) have been investigated, the majority of previous studies have focused on the transcriptional regulation of NEAT1 (10).

Posttranscriptional RNA N6-methyladenosine (m^6^A) is a reversible modification, in which the sixth nitrogen atom of adenosine is methylated. As a subtype of epitranscriptomic information, it has been extensively studied in various biological processes and disease settings (11, 12). The methyltransferase complex responsible for m^6^A deposition includes core components such as METTL3, METTL14, and WTAP. Transcripts carrying m^6^A modifications can be recognized by multiple reader proteins, including YTHDF1-YTHDF3 and YTHDC1-YTHDC2. Interestingly, the effects of m^6^A modifications depend on the specific readers (13, 14). For instance, YTHDF2 recognizes m^6^A and contributes to RNA decay (15). To date, two m^6^A demethylases, FTO and ALKBH5, have been identified (16, 17). Among them, ALKBH5 belongs to the AlkB subfamily of the Fe (II)/2-oxoglutarate dioxygenase superfamily (17). Its dynamics in abundance, subcellular localization, activity, and so on may be critical for the selective m^6^A demethylation of context-dependent substrates.

We previously found that ALKBH5 expression is induced under hypoxic conditions, which promotes m^6^A demethylation on NEAT1-2 (thereafter as NEAT1 unless otherwise specified), ultimately preventing its decay by dissociating YTHDF2 from the transcript. Thus, stabilized NEAT1 facilitates PS assembly (18). However, it is yet uncertain how m^6^A demethylation of NEAT1 is prioritized, given the impaired demethylase activity at the deprivation of oxygen.

In this study, we have confirmed that ALKBH5 forms phase-separated droplets through its C-terminal intrinsically disordered region (cIDR) and is incorporated into PS. This spatial enrichment of ALKBH5 in response to hypoxia ensures rapid and sufficient m^6^A demethylation of NEAT1, stabilizing the transcript and facilitating PS assembly. Consequently, the ALKBH5-cIDR-mediated phase separation contributes to a positive feedback loop between incorporation into PS and NEAT1 demethylation, which fosters PS assembly and cancer cell adaptation to hypoxia.

## Results

### ALKBH5 specifically interacts with proteins in paraspeckles

**Figure 1:**
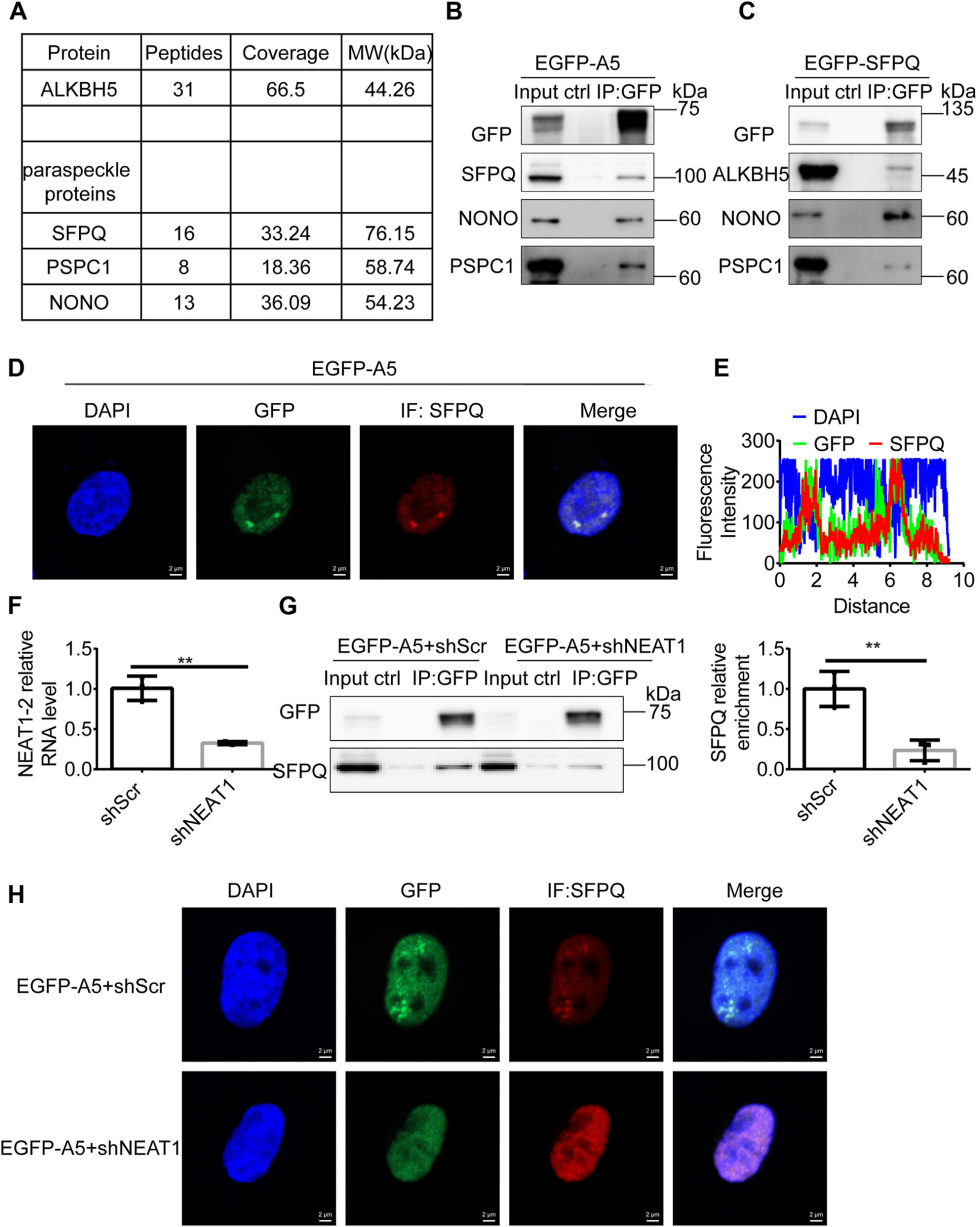
**ALKBH5 interacts with proteins in paraspeckles.***A*, identification of ALKBH5-interacting protein by mass spectrometry. The list of proteins specifically identified in HA affinity purifications followed by mass spectrometry analysis in HA-ALKBH5-overexpressed 293T cells. The number of unique peptides, coverage of the whole protein, and the molecular weight (MW, kDa) are shown. *B* and *C*, immunoprecipitation (IP) using the anti-GFP affinity gel, respectively, in EGFP-ALKBH5 (thereafter short as EGFP-A5) (*B*) or EGFP-SFPQ (*C*) overexpressed U87 cells, followed by Western blot analysis with designated antibodies. Protein A/G beads served as a negative control (ctrl). *D* and *E*, SFPQ immunostaining was performed in EGFP-ALKBH5-overexpressed U87 cells. Representative images are shown (*D*). The scale bars represent 2 μm. The fluorescence intensity curves are shown (*E*). *F*, NEAT1 RNA levels in U87 cells transduced with shRNA construct targeting NEAT1 (shNEAT1) or scramble shRNA (shScr) were determined by real-time quantitative PCR. Two-tailed unpaired Student’s *t* test. ∗∗, *p* < 0.01, n = 3. *G*, *left panel*: GFP pull-down to compare the interaction between ALKBH5 and SFPQ in NEAT1-deficient or control U87 cells overexpressing EGFP-ALKBH5. Protein A/G beads served as a negative control (ctrl). *Right panel*: statistical analysis to compare the relative SFPQ enrichment in the two groups (n = 3). *H*, immunofluorescence of SFPQ in NEAT1-deficient or control U87 cells overexpressing EGFP-ALKBH5. The scale bars represent 2 μm.

To better understand the mechanistic roles of ALKBH5, we conducted an affinity purification and mass spectrometry (MS) analysis to identify the proteins that are associated with ALKBH5. Briefly, we expressed HA-tagged ALKBH5 (HA-ALKBH5) in 293T cells, prepared nuclear extracts, and subjected them to affinity purification using an anti-HA antibody. MS analysis indicates that all core members of PS proteins, including SFPQ, NONO, and PSPC1, are significantly enriched in the copurified fractions (Fig. 1*A*). We further confirmed these protein–protein interactions by performing anti-GFP immunoprecipitation (IP) in the EGFP-ALKBH5 stably expressed human glioblastoma cell line U87 (Fig. 1*B*). Similarly, ectopically expressed EGFP-SFPQ in U87 cells pulls down ALKBH5, although it more efficiently pulls down NONO (Fig. 1*C*). In addition, the immunofluorescence (IF) assay shows a colocalization of SFPQ with EGFP-ALKBH5, mainly in the form of intranuclear granules (Fig. 1, *D* and *E*). Hence, ALKBH5 not only functions upstream of NEAT1 for PS assembly but also likely exists in PS.

To confirm the intranuclear granules observed in the previous experiments are indeed PS and the interaction is dependent on PS formation, we depleted NEAT1 in EGFP-ALKBH5 U87 cells through specific shRNA (shNEAT1) (Fig. 1*F*). As shown in Figure 1*G*, GFP-ALKBH5 pulls down significantly less SFPQ in NEAT1-deficient cells compared with the control (Fig. 1*G*). And the intranuclear ALKBH5 and SFPQ condensates fail to be observed at the absence of NEAT1 by the IF assay (Fig. 1*H*). These data illustrate that overexpressed ALKBH5 condensates with PS proteins in PS.

### ALKBH5 phase separates through its C-terminal IDR *in vitro*

**Figure 2:**
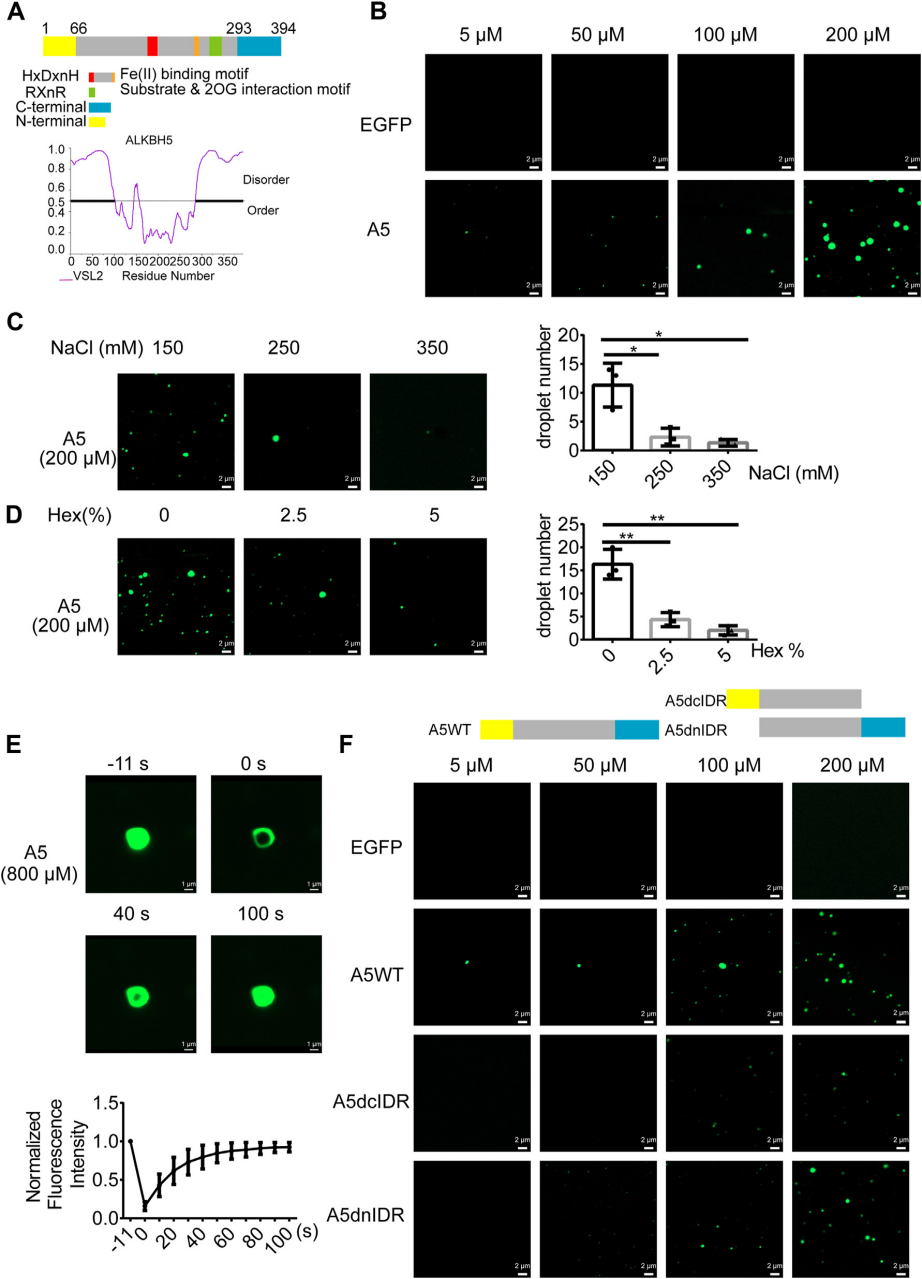
**ALKBH5 phase separates through its C-terminal intrinsically disordered region (IDR) *in vitro*.***A*, *top panel*: schematic diagram of the ALKBH5 structure. *Bottom panel*: prediction of ALKBH5 IDRs using PONDR. Disordered amino acids are highlighted in black. *B*, representative confocal images of droplet formation experiments with EGFP-ALKBH5 protein concentration gradients. EGFP alone is included as a control. All of the assays were performed in the presence of 150 mM NaCl, and 10% PEG-6000 was used as a crowding agent. The scale bars represent 2 μm. *C* and *D*, left panel: representative confocal images of EGFP-ALKBH5 droplets treated with different concentrations of NaCl (*C*) or 1,6-hexanediol (*D*). All experiments were performed in the presence of 200 μM EGFP-ALKBH5 and 150 mM NaCl, and 10% PEG-6000 was used as a crowding agent. The scale bars represent 2 μm. *Right panel*: statistical analysis to compare droplet numbers at each condition (n = 3). *E*, fluorescence recovery after photobleaching of EGFP-ALKBH5 droplets. Representative images are shown in the *top panel*. The protein concentration was 800 μM. Droplet formation assays were performed with protein concentration 800 μM in the presence of 150 mM NaCl and 10% PEG-6000. The scale bars represent 1 μm. Quantitative analysis of the average recovery of fluorescence of EGFP-ALKBH5 is shown in the *bottom panel*. Data are presented as mean ± SEM (n = 5). *F*, *top panel*: schematic diagram of the full length or truncates of EGFP-ALKBH5 proteins. *Bottom panel*: representative confocal images of a concentration series of droplet formation assays testing droplet formation of the full length or truncates of EGFP-ALKBH5 proteins. EGFP alone is included as a control. All of the assays were performed in the presence of 150 mM NaCl and 10% PEG-6000. The scale bars represent 2 μm.

To find out the biochemical basis of ALKBH5 condensation in PS, we conducted a thorough structural analysis of the protein. The catalytic domain of ALKBH5, consisting of amino acids 74 to 294 with a distorted double-stranded beta-helix fold and conserved 2-oxoglutarate and ferrous ion binding sites (19), has been well characterized. However, the N and C termini of the protein have not been extensively studied. Using the Predictor of Natural Disordered Regions (PONDR), we identified these regions as intrinsically disordered regions (IDRs) (Fig. 2*A*).

IDRs are characterized by low sequence complexity and often form reversible biomolecular condensates in a process called liquid–liquid phase separation (LLPS) (20, 21). Thus, we hypothesized that ALKBH5 might have the ability of forming condensates in aqueous solution. To test this hypothesis, we expressed and purified recombinant GST-EGFP-ALKBH5-WT from *Escherichia coli*. By adjusting the protein to different concentrations in the buffer with titrated salt or fatty alcohol, we performed the droplet formation assay. As shown in Figure 2*B*, the droplets formed by EGFP-ALKBH5, rather than EGFP alone, become larger and the number of droplets increases with elevated protein concentrations. And treatment of EGFP-ALKBH5 condensates (protein concentration 200 μM) with increasing concentrations of NaCl or 1, 6-hexanediol reduces the number of condensates (Fig. 2, *C* and *D*). Additionally, we utilized the fluorescence recovery after photobleaching assay to test ALKBH5 protein fluidity. As shown in Figure 2*E*, the fluorescence recovers from the bleached region in a few minutes, suggesting that ALKBH5 does undergo phase separation *in vitro*.

To find out which IDR of ALKBH5 is responsible for mediating phase separation, we generated two truncated mutant constructs from the wildtype (WT) of ALKBH5: the N-terminal IDR-deletion mutant (dnIDR) and the C-terminal IDR-deletion mutant (dcIDR) (Fig. 2*F*). Our results indicate that the cIDR deletion specifically affects the droplet formation at titrated protein concentrations. Therefore, we conclude that ALKBH5 promotes LLPS through its cIDR *in vitro*.

### ALKBH5 phase separates and incorporates into PS through its cIDR

**Figure 3:**
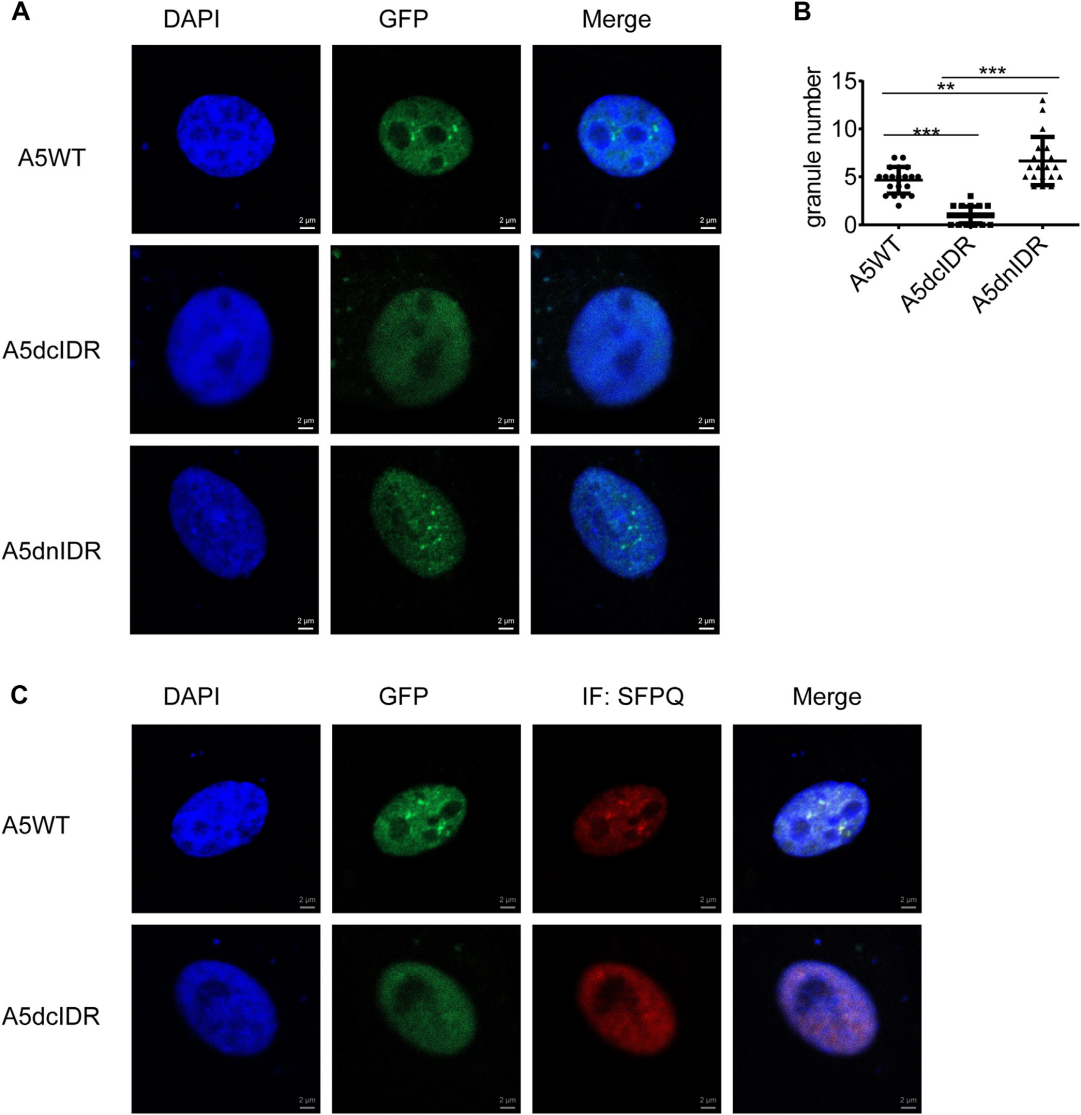
**ALKBH5 phase separates and incorporates into paraspeckles through its C-terminal intrinsically disordered region (cIDR).***A*, representative confocal images of U87 cells expressing the full length or truncates of EGFP-ALKBH5 proteins. The scale bars represent 2 μm. *B*, the paraspeckle numbers were counted for 20 corresponding U87 cells. Two-tailed unpaired Student’s *t* test. ∗∗*p* < 0.01, ∗∗∗*p* < 0.001. *C*, SFPQ immunostaining was performed in U87 cells expressing EGFP-A5WT or EGFP-A5dcIDR. The scale bars represent 2 μm.

To further determine whether the cIDR of ALKBH5 is responsible for nuclear condensation in living cells, we compared the GFP signals of EGFP-ALKBH5-WT or the two truncation mutants in U87 cells. As shown in Figure 3*A* and *B*, EGFP-ALKBH5-WT and dnIDR form multiple granules in the nucleus. In contrast, significantly fewer granules were observed in the dcIDR groups of cells. Additionally, unlike ALKBH5-WT condensation with SFPQ in PS (Figs. 1*H* and 3*C*), overexpressed ALKBH5-cIDR fails to assemble granules with SFPQ (Fig. 3*C*). Therefore, the LLPS capability of ALKBH5 is indispensable for PS formation.

### Rapid ALKBH5 condensation is coupled with m^6^A demethylation of NEAT1 and PS assembly in response to hypoxia

**Figure 4:**
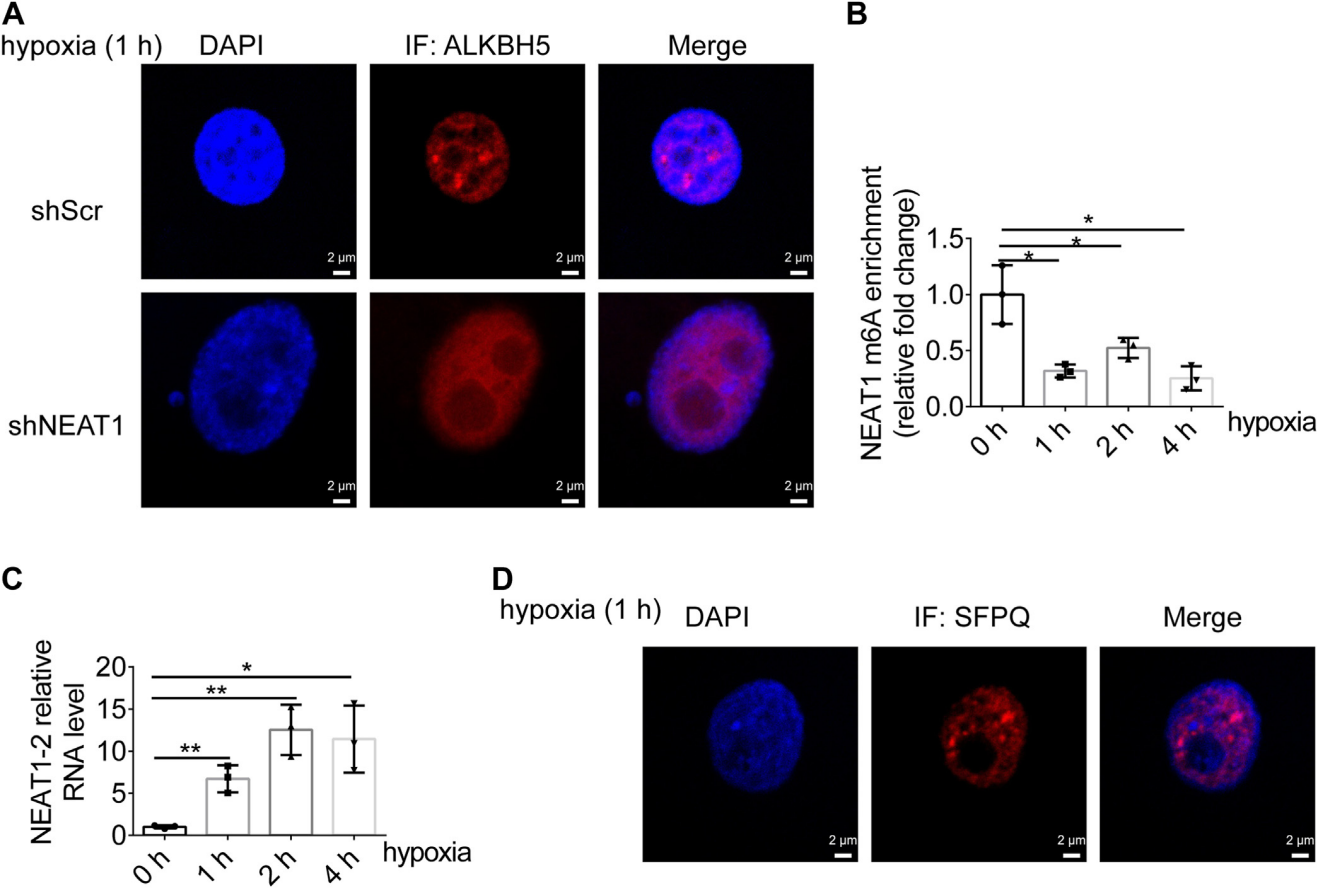
**Rapid ALKBH5 condensation is coupled with m**^**6**^**A demethylation of NEAT1 and paraspeckles assembly in response to hypoxia.***A*, ALKBH5 immunostaining was performed in NEAT1-deficient or control U87 cells after 1 h of hypoxia treatment. Representative images are shown. The scale bars represent 2 μm. *B*, MeRIP (m^6^A-IP)-qPCR analysis of the NEAT1 m^6^A peak region in U87 cells at different time points of hypoxia treatment. Two-tailed unpaired Student’s *t* test. ∗*p* < 0.05, n = 3. *C*, real-time quantitative PCR analysis of the RNA expression levels of NEAT1-2 in U87 cells at different time points of hypoxia treatment. Two-tailed unpaired Student’s *t* test. ∗, *p* < 0.05, ∗∗, *p* < 0.01, n = 3. *D*, SFPQ immunostaining was performed in U87 cells after 1 h of hypoxia treatment. Representative images are shown. The scale bars represent 2 μm.

**Supporting Figure S1:**
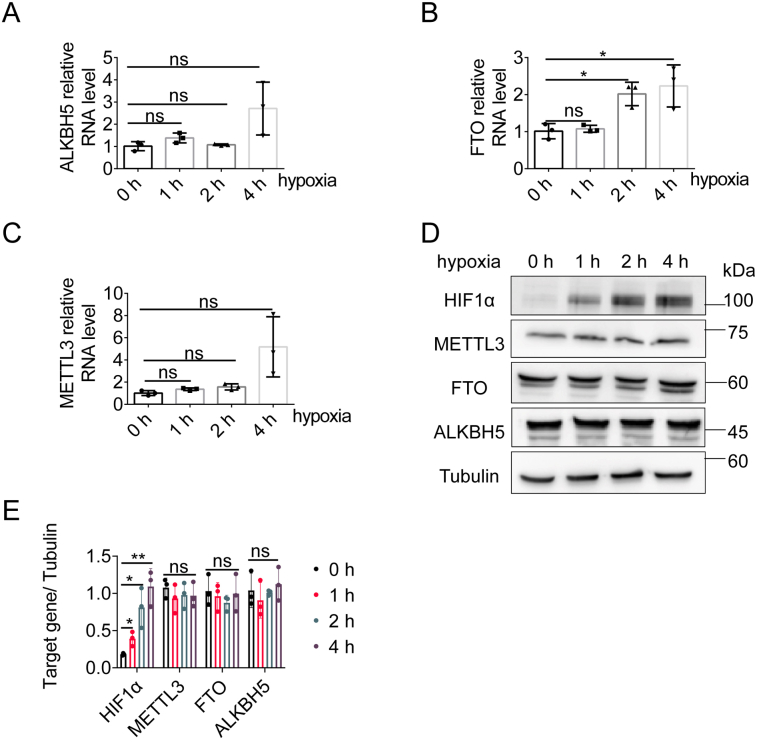


We previously thought that hypoxia-induced ALKBH5 expression was responsible for NEAT1 stability and subsequent PS assembly (18). However, considering the propensity of ALKBH5 to promote phase separation and PS formation, we wondered whether ALKBH5 condensation and PS assembly might occur prior to upregulated expression of ALKBH5 under hypoxic conditions. Indeed, IF assays demonstrate that ALKBH5 forms condensates within 1 h of hypoxia treatment (Fig. 4*A*). Actually, short-term hypoxia exposure (within 4 h) does not affect either the mRNA or protein levels of ALKBH5, FTO, or METTL3 (Fig. S1, *A*–*D*), but it does trigger rapid m^6^A demethylation of NEAT1, as evidenced by m^6^A RNA-immunoprecipitation-qPCR analysis (Fig. 4*B*). Accordingly, NEAT1 expression levels increase significantly and PS assembly is augmented within 1 h of hypoxia treatment (Fig. 4, *C* and *D*). Furthermore, the depletion of NEAT1 prevents the induction of ALKBH5 condensation (Fig. 4*A*). Together, these data suggest that ALKBH5 is rapidly integrated into PS, which in turn facilitates m^6^A demethylation of NEAT1 and PS assembly in response to hypoxia. ALKBH5-mediated phase separation may be the underlying mechanism for these interconnected processes.

### ALKBH5-cIDR is required for hypoxia-induced m^6^A demethylation of NEAT1 and PS assembly

**Figure 5:**
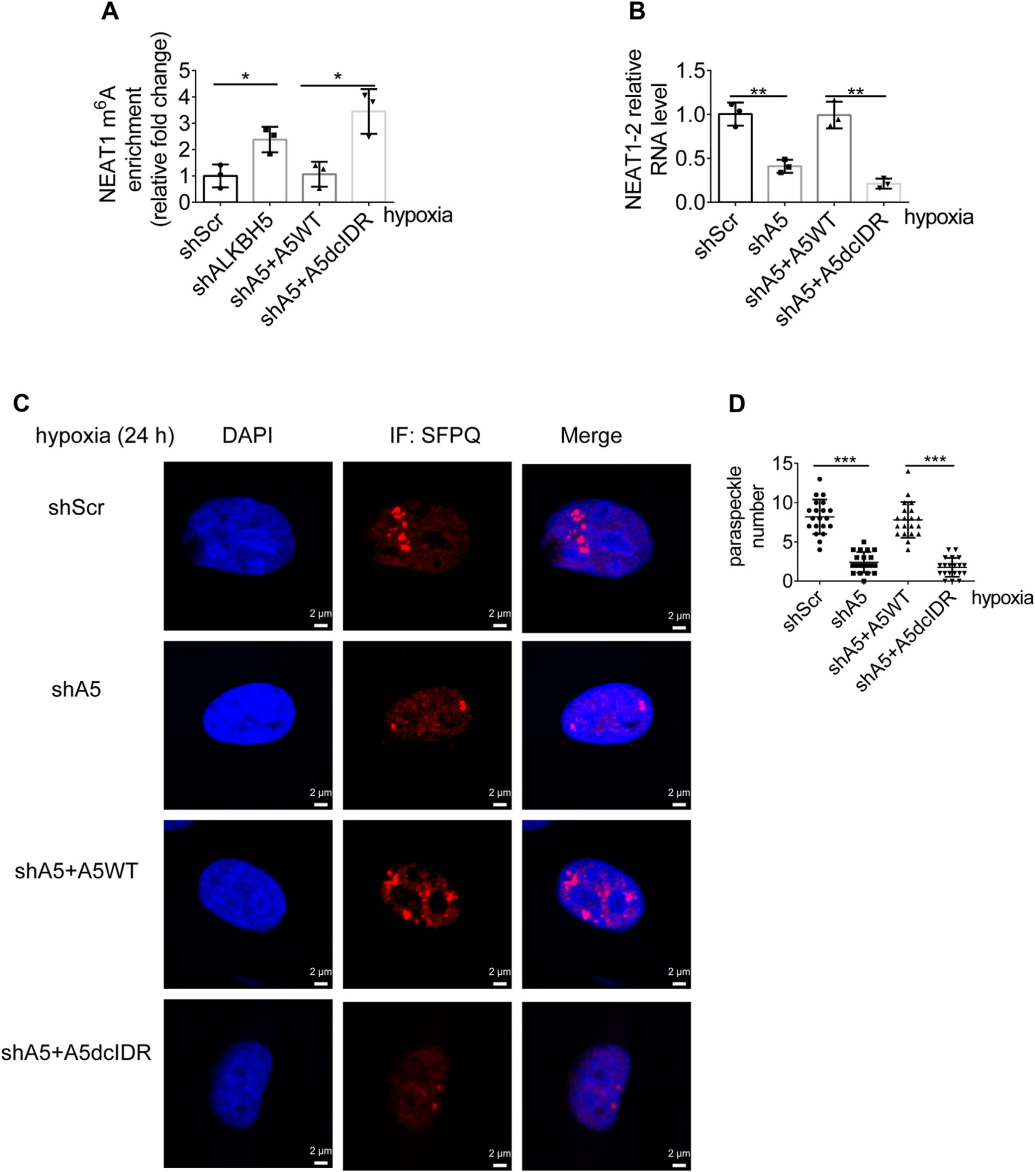
**ALKBH5-cIDR is required for hypoxia-induced m**^**6**^**A demethylation of NEAT1 and paraspeckles assembly.***A*, MeRIP (m^6^A-IP)-qPCR analysis of the NEAT1 m6A peak region in ALKBH5-deficient U87 cells rescued by A5-WT or dcIDR mutant under hypoxic conditions. Two-tailed unpaired Student’s *t* test. ∗*p* < 0.05, n = 3. *B*, real-time quantitative PCR analysis of RNA expression levels of NEAT1-2 in corresponding U87 cells under hypoxic conditions. Two-tailed unpaired Student’s *t* test, ∗∗, *p* < 0.01, n = 3. *C*, SFPQ immunostaining was performed in corresponding U87 cells under hypoxic conditions. Representative images are shown. The scale bars represent 2 μm. *D*, the number of paraspeckles was counted and analyzed in 20 corresponding U87 cells. Two-tailed unpaired Student’s *t* test, ∗∗∗*p* < 0.001.

To confirm the necessity of ALKBH5-cIDR in PS formation under physiological or pathophysiological conditions, we followed to find how ALKBH5-dcIDR affects NEAT1 demethylation in response to hypoxia. For the cell models, we generated ALKBH5 knockdown (shA5) U87 cells and re-expressed either ALKBH5-WT or dcIDR (shA5+A5WT or dcIDR). After exposure of these cells to hypoxia, the m^6^A levels of NEAT1 are significantly higher in ALKBH5-deficient (shA5 and shA5+A5dcIDR) than in ALKBH5-proficient (control and shA5+A5WT) cells (Fig. 5*A*). This indicates ALKBH5-dcIDR fails to demethylate m^6^A on NEAT1, although the catalytic domain remains intact. Accordingly, the NEAT1 expression levels are significantly lower in ALKBH5-deficient cells than in ALKBH5-proficient cells (Fig. 5*B*). Consistently, IF analysis of SFPQ demonstrates that ALKBH5 deficiency significantly affects hypoxia-induced PS assembly (Fig. 5, *C* and *D*). Therefore, ALKBH5 cIDR-mediated phase separation may foster positive feedback for hypoxia-induced NEAT1 stability and PS assembly.

**Supporting Figure S2:**
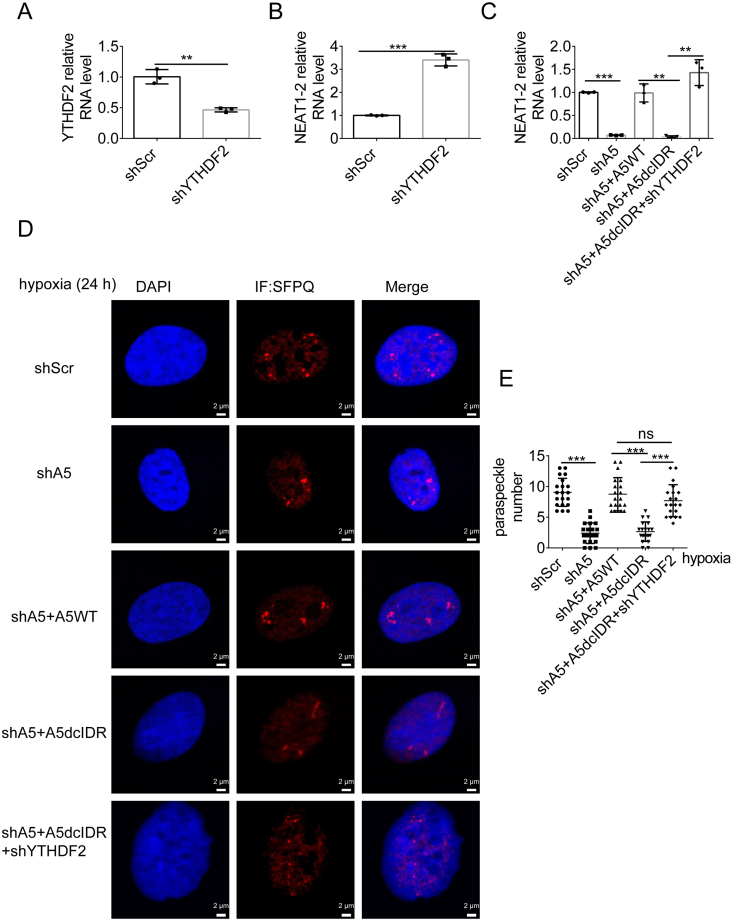


To further assess whether the destabilization of NEAT1 does account for impaired PS assembly in the ALKBH5-deficient cells, we generated the YTHDF2-deficient (shYTHDF2) U87 cells. As examined by real-time quantitative PCR (RT-qPCR) analysis, successful depletion of YTHDF2 (Fig. S2*A*) stabilizes NEAT1 (Fig. S2*B*). And the decreased NEAT1 expression in ALKBH5-dcIDR mutant cells is significantly reversed by depletion of YTHDF2 (Fig. S2*C*). Accordingly, PS numbers are restored when NEAT1 is stabilized by depletion of YTHDF2 in the ALKBH5-dcIDR cells (Fig. S2, *D* and *E*). These data indicate that ALKBH5-cIDR-mediated phase separation facilitates hypoxia-induced PS assembly by stabilizing NEAT1. Nevertheless, further studies are warranted to clarify the precise spatiotemporal regulation of the feedback loop between ALKBH5 incorporation to PS and NEAT1 m^6^A demethylation.

### ALKBH5-cIDR is required for hypoxia-driven cancer cell invasion

**Figure 6:**
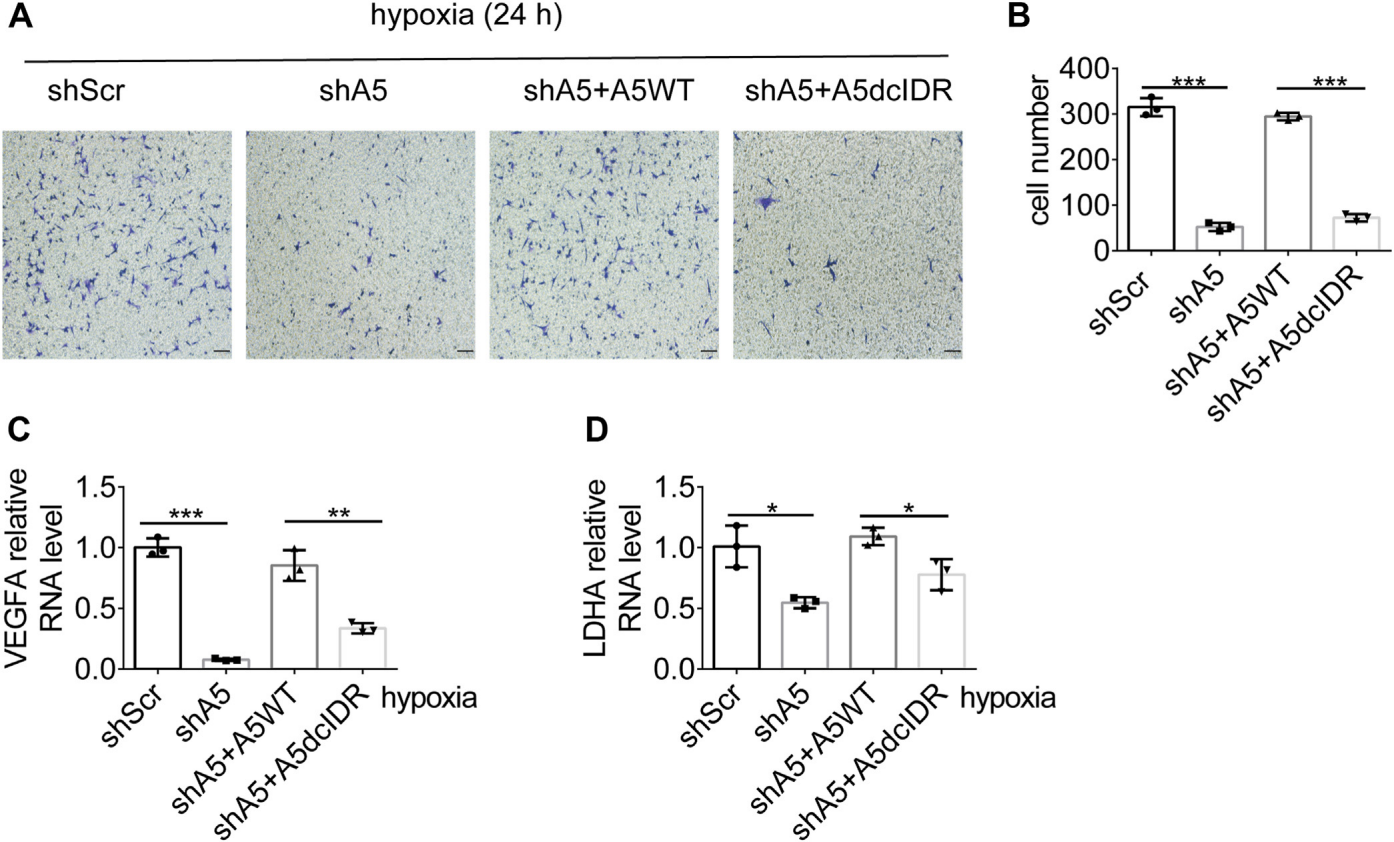
**ALKBH5-cIDR is required for hypoxia-induced effects.***A* and *B*, transwell assays (*A*) were performed to measure cIDR's ability to promote invasion in ALKBH5-deficient U87 cells under hypoxia. The scale bars represent 100 μm. The statistical chart of the invasion cells is given in (*B*). Two-tailed unpaired Student’s *t* test, ∗∗∗*p* < 0.001, n = 3. *C* and *D*, VEGFA RNA levels (*C*) and LDHA RNA levels (*D*) in corresponding U87 cells were determined by real-time quantitative PCR analysis. Two-tailed unpaired Student’s *t* test, ∗*p* < 0.05, ∗∗*p* < 0.01, ∗∗∗*p* < 0.001, n = 3. cIDR, C-terminal intrinsically disordered region.

To investigate the impact of ALKBH5-cIDR on tumor cell behavior in response to hypoxia, we assessed the invasion ability of U87 cells using a transwell assay. Our results showed that both ALKBH5 depletion and ALKBH5-dcIDR significantly suppressed cell invasion in response to hypoxia (Fig. 6, *A* and *B*). Furthermore, RT-qPCR analysis demonstrated that the expression levels of hypoxia signature genes *VEGFA* and *LDHA* were significantly downregulated in shA5 and shA5+A5-dcIDR cells compared with control or ALKBH5-WT restored cells (Fig. 6, *C* and *D*). These findings indicate that ALKBH5-cIDR is crucial for hypoxia-induced effects in cancer cells, including their invasion potential and gene expression profiles associated with hypoxia.

## Discussion

In recent years, emerging evidences have shown that numerous cellular processes are compartmentalized in biomolecular condensates, which are formed reversibly and dynamically by phase separation. These discrete assemblies of proteins and RNA molecules involved in similar cellular processes may facilitate specific biological functions or respond to extracellular cues (21, 22, 23, 24). Aberrant phase separation and transition have been linked to a variety of human diseases, including cancer (25, 26). Therefore, molecular mechanisms for the tight control of assembling and deassembling biomolecular condensates have caught huge interest.

**Figure 7:**
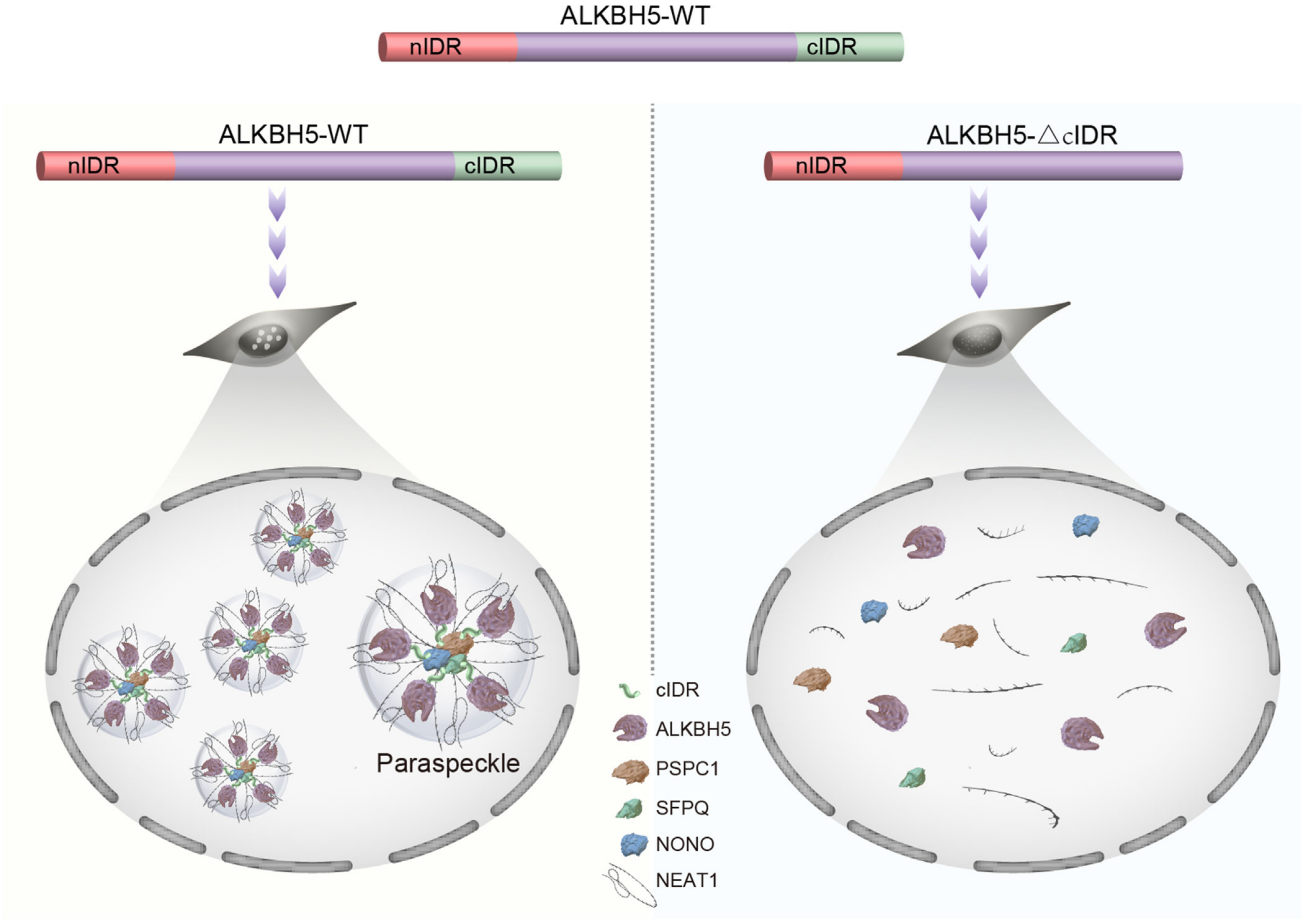
**A working model for ALKBH5-cIDR in phase separation and paraspeckle assembly.** In response to stress such as hypoxia, ALKBH5 undergoes condensation through its cIDR and facilitates paraspeckles assembly, in which NEAT1 m^6^A is demethylated and stabilized and therefore fosters a positive back. If the cIDR of ALKBH5 is truncated, it is inefficient to target NEAT1 for m^6^A demethylation, leading to NEAT1 instability and compromised paraspeckles assembly. cIDR, C-terminal intrinsically disordered region; nIDR, N-terminal intrinsically disordered region.

In this study, we re-evaluate the roles of ALKBH5 in the assembly of PS. Going beyond unidirectional regulation of NEAT1 demethylation (18, 27), we establish a feedback loop between ALKBH5 incorporation into PS through phase separation and NEAT1 stabilization for hypoxia-induced PS assembly. According to our model, the intrinsic biochemical property of ALKBH5-cIDR in phase separation is the prerequisite for PS assembly (Fig. 7). Interestingly, a few previous studies have demonstrated that increased concentrations of enzymes and substrates in condensates increase enzymatic reaction rates or expand reaction specificity (28, 29, 30). In this regard, the phase-separated compartment of ALKBH5 provides an attractive possibility for preferred m^6^A demethylation of key transcripts such as NEAT1 under conditions of inadequate demethylase activity. And our current data suggest that the rapid incorporation of ALKBH5 into PS is likely an initial event for hypoxia-induced PS assembly. However, it is still unclear how this process is triggered in response to stress such as hypoxia. Furthermore, the exact organization of ALKBH5 and PS components inside PS awaits to be determined. Thus, systematic studies are needed to determine which factors may either increase the LLPS capability or optimize the intracondensate organization in response to stress.

It is noteworthy that another m^6^A demethylase, FTO, has been found to interact with SFPQ and partly rely on it for demethylation substrate selection. However, no other PS components were identified as FTO-binding proteins (31). Thus, it is likely that FTO does not directly affect PS assembly. Although we demonstrate that the majority of interactions between ALKBH5 and PS protein occur in PS, we cannot exclude the possibility of their partnership outside of PS. Moreover, since all PS proteins possess PS-independent functions (31, 32, 33, 34, 35, 36), it is plausible that they also guide ALKBH5 to select demethylation substrates beyond NEAT1 for other cellular processes.

In summary, our study provides a novel insight into how ALKBH5-mediated phase separation contributes to preferential substrate demethylation. This mechanistic understanding offers targeting ALKBH5 condensation as a new anticancer strategy.

## Experimental procedures

### Cell culture

As described (37), 293FT cells and the human glioblastoma cell line U87 were grown in Dulbecco's modified Eagle's medium containing 10% fetal bovine serum (FBS). All cells were regularly authenticated by PCR and tested to be mycoplasma free. For the hypoxia treatment, cells were temporally cultured in hypoxic incubator with 5% CO_2_ and 1% O_2_.

### Plasmid construction and stable cell generation

Human ALKBH5 and its N-terminal or C-terminal IDR-deletion mutant or SFPQ cDNA were amplified and cloned into the pCDH-CMV-EGFP-MCS-EF1-Puro (Takara) according to the manufacturer’s protocol and verified by sequencing. Human ALKBH5 and its N-terminal or C-terminal IDR-deletion mutants were introduced into the pGEX6p-1-EGFP vector according to the protocol recommended by the Clone Express II One Step Cloning Kit (Vazyme C112). The cDNA of human ALKBH5 was introduced into pLenti-EF1-FLAG-HA vector and used for generation of stable cell lines. Specific oligonucleotides targeting YTHDF2 were cloned into pLKO.1 TRC cloning vector. The sequences for primers or oligos are listed in Table S1. Constructs for depleting ALKBH5 and NEAT1 were generated in previous studies (18).

### Complex purification and identification

To identify the proteins associated with ALKBH5, affinity purification was performed followed by MS analysis as described (38). Briefly, the nuclear extracts (100 mg) from the 293FT expressing HA-ALKBH5 were prepared as described previously. After the nuclear extracts were incubated with anti-HA-antibody overnight at 4 °C with rotation, 30 μl protein A/G beads (Smart-Lifesciences, SM01501) were added and incubated for 3 h. The beads were collected by centrifugation at 500*g* for 5 min and washed three times with cell lysis buffer (50 mM Tris-HCl, pH 7.4, 150 mM NaCl, 0.2 mM EDTA, 0.3% NP-40, 0.2 mM PMSF). The beads were then boiled in SDS loading buffer and run shortly into a SDS gel. A gel slice containing the purified proteins was isolated for MS analysis. The extracted tandem mass spectrometry data were processed using Mascot search engine. Tandem mass spectra were searched against SwissProt-Mouse database concatenated with reverse decoy database. Proteins containing unique mapped peptide with ion score meeting significant *p* value were considered.

### Immunoprecipitation

A total of 1 × 10^7^ cells were harvested and resuspended with cell lysis buffer followed by rotation at 4 °C for 30 min. After centrifugation at 12,000 rpm for 10 min, the supernatant was collected and incubated with GFP-beads (Smart-Lifesciences, SA070001) overnight at 4 °C. Ten percent of lysates was used as Input. On the next day, the beads were washed three times with cell lysis buffer and boiled in 1× SDS loading buffer, followed by Western blot analysis.

### Western blot

Hypoxia-treated cells were rapidly lysed with 1% SDS and boiled in SDS loading buffer and analyzed by Western blot assays. The following antibodies were used: anti-ALKBH5 (1:1000, Proteintech, 16837–1-AP), anti-HIF1a (1:1000, GeneTex, GTX127309), anti-FTO (1:1000, Abclonal A3861), anti-METTL3 (1:1000, Abclonal, A19079), anti-b-Tubulin (1:10,000, Abclonal, AC021), anti-SFPQ (1:1000, Abclonal, A3494), anti-NONO (1:1000, Abclonal, A3800), anti-PSPC1 (1:1000, Proteintech, 116714-1-AP), and anti-GFP (1:1000, Proteintech, 50430-2-AP). Direct-load Color Prestained Protein Marker (GenStar, M221) was used in all assays.

### Immunofluorescence

Cells were fixed with 1% formaldehyde, permeabilized and blocked at the same time with PBS containing 0.5% Triton X-100 and 3% FBS, and incubated overnight at 4 °C with the appropriate primary antibody. On the second day, the cells were incubated with TRITC secondary antibody (1:100, ZSGB-BIO, ZF-0316) for 2 h at room temperature and then mounted with Hoechst. The antibodies include anti-ALKBH5 (1:200, Proteintech, 16837–1-AP) and anti-SFPQ (1:200, Abclonal, A3494).

### Real-time quantitative PCR

Total RNA was isolated using TRIGene reagent (Genstar, P118-05) and subjected to reverse transcription with RevertAid Reverse Transcriptase (Thermo Scientific, EP0442). The RT-qPCR primer sequences are summarized in Table S1.

### MeRIP (m^6^A-IP)-qPCR

The MeRIP (m^6^A-IP)-qPCR was performed as described (18). Briefly, total RNA (5 μg) was isolated and fragmented into approximately 200-nt-long fragments in RNA fragmentation buffer (100 mM Tris-HCl, 100 mM ZnCl_2_ in nuclease-free H_2_O) at 70 °C for 5 min. Protein A/G magnetic beads were tumbled with 5 μg anti-m^6^A antibody (Abclonal, A9841) at 4 °C at least 6 h. Then, the antibody-bead mixture was resuspended in 500 μl of the IP reaction mixture containing fragmented total RNA, 500 μl IP buffer, and 5 μl RNase Inhibitor and incubated for 2 h at 4 °C. Then the RNA reaction mixture was washed in the low/high-salt washing buffer. The m^6^A-enriched RNA was eluted with 14 μl DEPC H_2_O according to the instructions of RNeasy Mini Kit (QIAGEN, 74106). Primers for MeRIP (m^6^A-IP)-qPCR are listed in Table S1.

### Recombinant protein purification

cDNAs were introduced into the pGEX6p-1-EGFP vector and sequenced to ensure sequence identity. The expression plasmids were then transformed into *E. coli* BL21. A fresh bacterial colony was inoculated into LB medium containing ampicillin and grown overnight at 37 °C. Then the bacterial culture was diluted 1:100 in 400 ml LB with freshly added ampicillin and grown at 37 °C, 220 rpm for 2.5 h. IPTG was added to 0.3 mM when *A*_600_ reached 0.6 to 0.8. After shaking at 16 °C, 160 rpm for 8 h, we harvested the bacteria and resuspended them in bacterial lysis buffer (50 mM Tris-HCl, PH 7.5, 100 mM NaCl, 1 mM DTT, 0.2 mM DTT, 1% TritonX-100). After ultrasonication, the protein supernatant was collected and incubated with Glutathione beads (Smart-Lifesciences, SA008025) overnight at 4 °C with rotation. The beads were then washed three times with bacterial lysis buffer. The protein was cleaved from the beads using cleavage enzyme HRV-3C.

### *In vitro* droplet formation assay

The droplet formation assay was performed as described (39, 40, 41). Briefly, Recombinant GFP fusion proteins were concentrated and desalted to an appropriate protein concentration and 150 mM NaCl using Amicon Ultra centrifugal filters (30 K MWCO, Millipore). Recombinant proteins were added to solutions at varying concentrations and 10% PEG-6000 as crowding agent in droplet formation buffer (50 mM Tris-HCl pH 7.5, 150 mM NaCl, 2 mM EDTA, 1 mM DTT). The protein solution was immediately loaded onto a homemade chamber comprising a glass slide with a coverslip attached by two parallel strips of double-sided tape. Slides were then imaged with confocal microscope. Unless indicated, images presented are of droplets settled on the glass coverslip.

### Fluorescence recovery after photobleaching assay

The droplets prepared above were photobleached by drawing a region of interest onto the droplets and then exposing the marked areas to intense 488-nm laser lines. For photobleaching, 100% power of the 488-nm solid-state laser line and 10 iterations were used.

### Invasion assay

Twenty-four-well transwell chambers with 8-mm pore size (Corning Costar, 3422) were used to perform migration assay as described (42). The upper chamber was covered with matrix gel and inoculated with 5 × 10^4^ U87 cells with serum-free medium. The lower chamber was filled with medium containing 10% FBS as a chemotaxis force. Cells in the upper chamber were carefully removed 24 h later. The invaded cells were washed with PBS and fixed with 4% formaldehyde for 10 min, stained with 0.1% crystal violet for 30 min at room temperature and imaged.

## Data availability

All data are available upon request. Sequences of shRNAs and primer oligonucleotides are provided in Table S1 in supporting information. The mass spectrometry proteomics data have been deposited to the ProteomeXchange Consortium *via* the PRIDE partner repository with the dataset identifier PXD042183.

## Supporting information

This article contains supporting information.

## Conflict of interest

The authors declare that they have no conflicts of interest with the contents of this article.

## References

[1] Fox A.H., Lam Y.W., Leung A.K., Lyon C.E., Andersen J., Mann M. (2002). Paraspeckles: a novel nuclear domain. Curr. Biol..

[2] Sasaki Y.T., Ideue T., Sano M., Mituyama T., Hirose T. (2009). MENepsilon/beta noncoding RNAs are essential for structural integrity of nuclear paraspeckles. Proc. Natl. Acad. Sci. U. S. A..

[3] Fox A.H., Lamond A.I. (2010). Paraspeckles. Cold Spring Harb. Perspect. Biol..

[4] McCluggage F., Fox A.H. (2021). Paraspeckle nuclear condensates: global sensors of cell stress?. Bioessays.

[5] Clemson C.M., Hutchinson J.N., Sara S.A., Ensminger A.W., Fox A.H., Chess A. (2009). An architectural role for a nuclear noncoding RNA: NEAT1 RNA is essential for the structure of paraspeckles. Mol. Cell.

[6] Yamazaki T., Souquere S., Chujo T., Kobelke S., Chong Y.S., Fox A.H. (2018). Functional domains of NEAT1 architectural lncRNA induce paraspeckle assembly through phase separation. Mol. Cell.

[7] Modic M., Grosch M., Rot G., Schirge S., Lepko T., Yamazaki T. (2019). Cross-regulation between TDP-43 and paraspeckles promotes pluripotency-differentiation transition. Mol. Cell.

[8] Morchikh M., Cribier A., Raffel R., Amraoui S., Cau J., Severac D. (2017). HEXIM1 and NEAT1 long non-coding RNA form a multi-subunit complex that regulates DNA-mediated Innate immune response. Mol. Cell.

[9] Hu S.B., Xiang J.F., Li X., Xu Y., Xue W., Huang M. (2015). Protein arginine methyltransferase CARM1 attenuates the paraspeckle-mediated nuclear retention of mRNAs containing IRAlus. Genes Dev..

[10] Choudhry H., Albukhari A., Morotti M., Haider S., Moralli D., Smythies J. (2015). Tumor hypoxia induces nuclear paraspeckle formation through HIF-2alpha dependent transcriptional activation of NEAT1 leading to cancer cell survival. Oncogene.

[11] Murakami S., Jaffrey S.R. (2022). Hidden codes in mRNA: control of gene expression by m(6)A. Mol. Cell.

[12] Zhao B.S., Roundtree I.A., He C. (2017). Post-transcriptional gene regulation by mRNA modifications. Nat. Rev. Mol. Cell Biol..

[13] Yang Y., Hsu P.J., Chen Y.S., Yang Y.G. (2018). Dynamic transcriptomic m(6)A decoration: writers, erasers, readers and functions in RNA metabolism. Cell Res..

[14] Shi H., Wei J., He C. (2019). Where, when, and how: context-dependent functions of RNA methylation writers, readers, and erasers. Mol. Cell.

[15] Wang X., Lu Z., Gomez A., Hon G.C., Yue Y., Han D. (2014). N6-methyladenosine-dependent regulation of messenger RNA stability. Nature.

[16] Jia G., Fu Y., Zhao X., Dai Q., Zheng G., Yang Y. (2011). N6-methyladenosine in nuclear RNA is a major substrate of the obesity-associated FTO. Nat. Chem. Biol..

[17] Zheng G., Dahl J.A., Niu Y., Fedorcsak P., Huang C.M., Li C.J. (2013). ALKBH5 is a mammalian RNA demethylase that impacts RNA metabolism and mouse fertility. Mol. Cell.

[18] Dong F., Qin X., Wang B., Li Q., Hu J., Cheng X. (2021). ALKBH5 facilitates hypoxia-induced paraspeckle assembly and IL8 secretion to generate an Immunosuppressive tumor Microenvironment. Cancer Res..

[19] Xu C., Liu K., Tempel W., Demetriades M., Aik W., Schofield C.J. (2014). Structures of human ALKBH5 demethylase reveal a unique binding mode for specific single-stranded N6-methyladenosine RNA demethylation. J. Biol. Chem..

[20] Zhang H., Ji X., Li P., Liu C., Lou J., Wang Z. (2020). Liquid-liquid phase separation in biology: mechanisms, physiological functions and human diseases. Sci. China Life Sci..

[21] Shin Y., Brangwynne C.P. (2017). Liquid phase condensation in cell physiology and disease. Science.

[22] Hnisz D., Shrinivas K., Young R.A., Chakraborty A.K., Sharp P.A. (2017). A phase separation model for transcriptional control. Cell.

[23] Boeynaems S., Alberti S., Fawzi N.L., Mittag T., Polymenidou M., Rousseau F. (2018). Protein phase separation: a new phase in cell biology. Trends Cell Biol..

[24] Sabari B.R., Dall'Agnese A., Young R.A. (2020). Biomolecular condensates in the Nucleus. Trends Biochem. Sci..

[25] Boija A., Klein I.A., Young R.A. (2021). Biomolecular condensates and cancer. Cancer Cell.

[26] Chandra B., Kriwacki R. (2022). Charting the human disease condensate dysregulome. Dev. Cell.

[27] Zhang J., Guo S., Piao H.Y., Wang Y., Wu Y., Meng X.Y. (2019). ALKBH5 promotes invasion and metastasis of gastric cancer by decreasing methylation of the lncRNA NEAT1. J. Physiol. Biochem..

[28] Sang D., Shu T., Pantoja C.F., Ibanez de Opakua A., Zweckstetter M., Holt L.J. (2022). Condensed-phase signaling can expand kinase specificity and respond to macromolecular crowding. Mol. Cell.

[29] Peeples W., Rosen M.K. (2021). Mechanistic dissection of increased enzymatic rate in a phase-separated compartment. Nat. Chem. Biol..

[30] Seif E., Kang J.J., Sasseville C., Senkovich O., Kaltashov A., Boulier E.L. (2020). Phase separation by the polyhomeotic sterile alpha motif compartmentalizes Polycomb group proteins and enhances their activity. Nat. Commun..

[31] Song H., Wang Y., Wang R., Zhang X., Liu Y., Jia G. (2020). SFPQ is an FTO-binding protein that facilitates the demethylation substrate preference. Cell Chem. Biol..

[32] Mitobe Y., Iino K., Takayama K.I., Ikeda K., Suzuki T., Aogi K. (2020). PSF promotes ER-positive breast cancer progression via posttranscriptional regulation of ESR1 and SCFD2. Cancer Res..

[33] Imamura K., Imamachi N., Akizuki G., Kumakura M., Kawaguchi A., Nagata K. (2014). Long noncoding RNA NEAT1-dependent SFPQ relocation from promoter region to paraspeckle mediates IL8 expression upon immune stimuli. Mol. Cell.

[34] Yasuhara T., Xing Y.H., Bauer N.C., Lee L., Dong R., Yadav T. (2022). Condensates induced by transcription inhibition localize active chromatin to nucleoli. Mol. Cell.

[35] Ma C., Karwacki-Neisius V., Tang H., Li W., Shi Z., Hu H. (2016). Nono, a bivalent domain factor, regulates Erk signaling and mouse Embryonic stem cell pluripotency. Cell Rep..

[36] Huang X., Bashkenova N., Hong Y., Lyu C., Guallar D., Hu Z. (2022). A TET1-PSPC1-Neat1 molecular axis modulates PRC2 functions in controlling stem cell bivalency. Cell Rep..

[37] Dong F., Li Q., Yang C., Huo D., Wang X., Ai C. (2018). PRMT2 links histone H3R8 asymmetric dimethylation to oncogenic activation and tumorigenesis of glioblastoma. Nat. Commun..

[38] Huo D., Yu Z., Li R., Gong M., Sidoli S., Lu X. (2022). CpG island reconfiguration for the establishment and synchronization of polycomb functions upon exit from naive pluripotency. Mol. Cell.

[39] Zamudio A.V., Dall'Agnese A., Henninger J.E., Manteiga J.C., Afeyan L.K., Hannett N.M. (2019). Mediator condensates localize signaling factors to key cell identity genes. Mol. Cell.

[40] Nott T.J., Petsalaki E., Farber P., Jervis D., Fussner E., Plochowietz A. (2015). Phase transition of a disordered nuage protein generates environmentally responsive membraneless organelles. Mol. Cell.

[41] Tatavosian R., Kent S., Brown K., Yao T., Duc H.N., Huynh T.N. (2019). Nuclear condensates of the Polycomb protein chromobox 2 (CBX2) assemble through phase separation. J. Biol. Chem..

[42] Lv X., Li Q., Liu H., Gong M., Zhao Y., Hu J. (2022). JUN activation modulates chromatin accessibility to drive TNFalpha-induced mesenchymal transition in glioblastoma. J. Cell. Mol. Med..

