# Supplementary material for: The disordered C terminus of ALKBH5 promotes phase separation and paraspeckles assembly
Source: J Biol Chem. 2023 Jul 18;299(8):105071. doi: 10.1016/j.jbc.2023.105071 (PMC10457456; doi:10.1016/j.jbc.2023.105071)
Supplement: Supporting Information [file mmc2.docx]

**Supplementary Figures S1–S2 Legends**

**Figure S1**

**A, B and C.** RT-qPCR analysis of the RNA expression levels of ALKBH5 **(A),** FTO **(B)** or METTL3 **(C)** in U87 cells at different hypoxia treatment time points. Two-tailed unpaired student’s *t*-test, ns, nonsignificant, *, *P*<0.05, n=2.

**D.** ALKBH5, FTO and METTL3 protein levels in U87 cells in different hypoxia treatment time points were determined by western blot analysis. Tubulin served as a loading control.

**Figure S2**

**A-B.** RT-qPCR analysis of YTHDF2 (**A**) or NEAT1–2 RNA levels (**B**) in YTHDF2 deficient or control U87 cells. Two-tailed unpaired student’s t-test. **, *P*<0.01, n=2.

**C.** RT-qPCR analysis of NEAT1–2 levels in corresponding U87 cells. Two-tailed unpaired student’s *t*-test. ns, nonsignificant, **, *P*<0.01, n=2.

**D.** SFPQ immunostaining was performed in corresponding U87 cells under hypoxic conditions. Representative images are shown. Scale bars, 2 μm.

**E.** PS numbers were counted and analyzed in 20 corresponding U87 cells. Two-tailed unpaired student’s *t*-test, ***, *P*<0.001.
